# Retinal oxygen metabolic function in choroideremia and retinitis pigmentosa

**DOI:** 10.1007/s00417-024-06659-8

**Published:** 2024-10-12

**Authors:** Dominique Prétot, Maria della Volpe Waizel, Karolina Kaminska, Philippe Valmaggia, Giorgio Placidi, Benedetto Falsini, Fabian N. Fries, Nóra Szentmáry, Carlo Rivolta, Hendrik P. N. Scholl, Giacomo Calzetti

**Affiliations:** 1 Department of Ophthalmology, University Hospital Basel, University of Basel, Basel, Switzerland; 2 Heuberger Eye Clinic, Olten, Switzerland; 3 Dr. Rolf M. Schwiete Center for Limbal Stem Cell and Congenital Aniridia Research, Saarland University, Homburg, Saar Germany; 4 Institute of Molecular and Clinical Ophthalmology Basel, Basel, Switzerland; 5 Department of Biomedical Engineering, University of Basel, Basel, Switzerland; 6 Ophthalmology Unit, Fondazione Policlinico Universitario ‘‘A. Gemelli’’ IRCCS/Università Cattolica del S. Cuore, Rome, Italy; 7 Department of Ophthalmology, Saarland University Medical Center, Homburg, Saar Germany; 8 Department of Genetics and Genome Biology, University of Leicester, Leicester, LE1 7RH UK; 9 Vista Vision Eye Clinic, Brescia, Italy

**Keywords:** Choroideremia, Retinal oxygen metabolic function, Inherited retinal diseases, Retinal oximetry, Retinitis pigmentosa

## Abstract

**Purpose:**

To measure the retinal oxygen metabolic function with retinal oximetry (RO) in patients with choroideremia (CHM) and compare these findings with retinitis pigmentosa (RP) patients and controls.

**Methods:**

Prospective observational study including 18 eyes of 9 molecularly confirmed CHM patients (9♂; 40.2 ± 21.2 years (mean ± SD), 77 eyes from 39 patients with RP (15♀ 24♂; 45.6 ± 14.7 years) and 100 eyes from 53 controls (31♀ 22♂; 40.2 ± 13.4 years). Main outcome parameters were the mean arterial (A-SO_2_; %), venular (V-SO_2_; %) oxygen saturation, and their difference (A-V SO_2_; %) recorded with the oxygen saturation tool of the Retinal Vessel Analyzer (IMEDOS Systems UG, Germany). Statistical analyses were performed with linear mixed-effects models.

**Results:**

Eyes suffering from CHM differed significantly from both RP and control eyes, when the retinal oxygen metabolic parameters were taken into account. While RP showed significantly higher A-SO_2_ and V-SO_2_ values when compared to controls, CHM showed opposite findings with significantly lower values when compared to both RP and controls (P < 0.001). The A-V SO_2_, which represents the retinal oxygen metabolic consumption, showed significantly lower values in CHM compared to controls.

**Conclusion:**

The retina in CHM is a relatively hypoxic environment. The decrease in oxygen levels may be due to the profound choroidal degeneration, leading to decreased oxygen flux to the retina. RO measurements may help understand the pathogenesis of CHM and RP. These findings may provide useful details to inform the planning of clinical trials of emerging therapies for CHM.

**Key messages:**

***What was known before?***

- Retinal oxygen metabolic function measured with retinal oximetry (RO) shows significant alterations in patients with retinitis pigmentosa.

***What this study adds:***

- RO function in choroideremia is significantly altered when compared to controls.
- Furthermore, RO in choroideremia shows opposing findings within different oxygen metabolic parameters to those that were so far known for retinitis pigmentosa.
- By providing insights into the retinal oxygen metabolic mechanisms, RO can help understand the underlying pathophysiology in choroideremia.

**Supplementary Information:**

The online version contains supplementary material available at 10.1007/s00417-024-06659-8.

## Introduction

Spectrophotometric retinal oximetry (RO) is a non-invasive method to quickly obtain oxygen saturation levels in retinal vessels through simple fundus photography [1]. It has been proven to be a useful tool in inherited retinal diseases (IRDs) and other disorders by previous studies [2–10]. As proper oxygenation of the retina is essential to maintain normal visual function [11], any alterations leading to impaired oxygen metabolism may have a detrimental effect on visual performance.

Increasing research interest in IRDs has risen in the recent decade as it is the leading cause of visual loss for patients of working age in industrialized countries and one of the most important reasons for visual impairment in childhood [12]. Pathophysiological mechanisms underlying this heterogenous group of genetic diseases are slowly being understood and new therapeutic approaches are being investigated.

Among IRDs, the utility of RO has been demonstrated in patients with retinitis pigmentosa (RP) where a progressive loss of photoreceptors has been shown to lead to a lower consumption of oxygen and a decreased oxygen demand of the retina [2–7]. It is hypothesized that this leads to a secondary increase in vascular oxygen saturation as a result of cellular apoptosis which may explain the vascular oxygen alterations in RP [2–7].

To the best of our knowledge, RO has not been investigated in the monitoring of choroideremia (CHM) nor have oxygen saturation analyses been conducted in CHM up to this date. Choroideremia is a monogenetic X-linked IRD affecting the photoreceptors, the retinal pigment epithelium (RPE), and the choroid. The mutations causing the disease are in the *CHM* gene encoding the Rab escort protein 1 (REP1), which is responsible for intracellular vesicle trafficking. Clinically, CHM has the features of a rod-first disease, which means that loss of rod function predominates early in the disease course, similar to RP. However, more pronounced degeneration of the choroid is evident in CHM than is seen in typical RP [13–16].

The aim of our study was to explore the changes in RO in CHM compared to controls and RP patients.

## Methods

This prospective observational study was performed on a total of 195 eyes from 101 subjects: 18 eyes from 9 patients with molecularly confirmed CHM and 77 eyes from 39 patients with RP were compared to 100 eyes from 53 controls. The study participants were examined in a single Ophthalmology centre (University of Basel, Department of Ophthalmology, Switzerland). This study was approved by the local authorities (Ethics Commission of Central and Northern Switzerland, EKNZ Basel Switzerland) with a positive vote for a prospective observational study (trial number EKNZ BASEC 2020–00122).

The inclusion criteria for all patients and controls were: Caucasian origin, refractive error spherical equivalent of < 6 dioptres for either myopia or hyperopia. Study participants with ocular or systemic pathology that may influence the retinal vessel oximetry data were excluded from this study. Additional exclusion criteria were: fundus oximetry images with inadequate quality or expressed unwillingness to participate in the study. All research procedures were carried out in accordance with institutional guidelines and the Declaration of Helsinki. Written informed consent was obtained before the examination. All participants underwent a detailed ophthalmic examination including refraction, best-corrected Snellen visual acuity, slit lamp examination, biomicroscopy, and fundoscopy. All CHM and RP patients underwent all required standard evaluations by experienced fellowship-trained retina specialists (G.C., M.d.V.W. and H.P.N.S.) depending on the clinical picture and diagnostic findings including, among others, electrophysiological testing, short-wavelength fundus autofluorescence and optical coherence tomography (OCT) imaging, visual field testing with semi-automated kinetic perimetry (background illumination 10 cd/m^2^, V4e, III4e, I4e, III3e isopters tested with the Octopus 900®), microperimetry and genetic testing. Controls were defined as healthy participants presenting no ophthalmic or systemic pathology.

Prior to RO measurements, both pupils were maximally dilated with Tropiphen eye drops (prepared in our institutional pharmacy as a combination of tropicamide 0.5% and phenylephrine 1%). Three drops per eye at 10-min intervals were applied.

### Retinal vessel oximetry acquisition

**Fig. 1:**
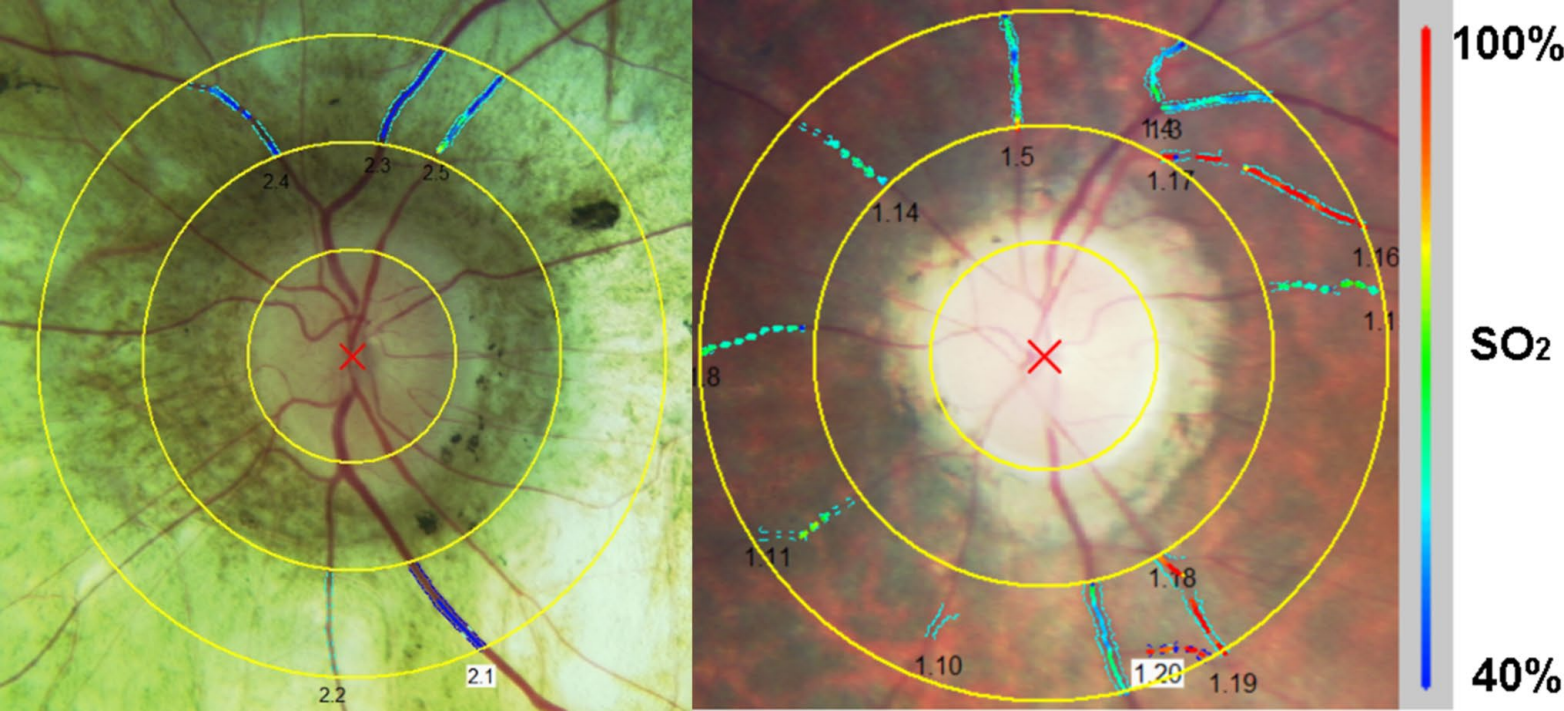
Example of a vessel map in the left eye of a patient suffering from choroideremia (left side) and a patient with retinitis pigmentosa (right side). Oxygen saturation was measured in all main retinal arterioles and venules within 1.0–1.5 optic disc diameter distance from the optic disc margin

A spectrophotometric oximetry unit for retinal vessel oximetry was used (IMEDOS Systems UG, Jena, Germany; Fundus camera FF450, Carl Zeiss Meditec AG, Jena, Germany). Fundus images were acquired using a camera system, DCC Digital Camera KY-F75 (JVC Inc., Yokohama, Japan) connected to the Zeiss fundus camera from a 50 degrees camera angle. The software operating the system (VISUALIS; IMEDOS Systems UG) differentiates simultaneously between oxygenated and deoxygenated haemoglobin based on different light imaging characteristics at different wavelengths, measuring the oxygen saturation level in the examined retinal vessel segment. In summary, RO imaging is performed at two different wavelengths: the green channel (548 ± 10 nm) measures the oxygen-insensitive image and the red channel (610 ± 10 nm) measures the oxygen-sensitive image [17, 18]. An optic disc-centred image protocol is used with two concentric rings in the peripapillary area: the inner with a radius of 1.0 optic disc diameters, the outer with a radius of 1.5 optic disc diameters. The region between both circles is defined as the area of interest where all measurements are performed (Fig. 1). Four test–retest fundus images for each eye were obtained [19]. Only those with optimal illumination, red channel illumination < 160 step of the scale, and green channel illumination > 60 step of the scale, were further selected for analysis. For RO analyses all main arterioles and venules were selected manually within the measurement area. The global mean oxygen saturation in retinal arterioles (A-SO_2_) and venules (V-SO_2_) was measured and subsequently their difference, the A-V SO_2_ representing an index of the retinal oxygen metabolic consumption, was calculated (Fig. 1).

### Statistical analysis

Primary study outcomes were: the mean arterial (A-SO_2_; %) and venous (V-SO_2_; %) oxygen saturation, and the difference between both (A-V SO_2_; %).

Before statistical evaluation, a normal distribution of all parameters was ensured with histograms and Shapiro–Wilk tests. In order to compare the subgroups, ANOVA with all pairwise comparisons was performed and all presented p-values were Bonferroni adjusted. To exclude age differences we calculated unpaired t-tests for all parameters of each group for female and male participants. Due to the genetic background of CHM there were only male participants in the corresponding group.

ANOVA-based linear mixed-effects models were calculated with SPSS® (IBM SPSS Statistics®, Version 29.0.2.0 including the advanced statistics package) which allows taking the dependency of the left and right eye in the same subject into account and is suitable for repeated measurements. To predict the effect of the different subgroups on oximetry estimations, the eye, the refractive spherical equivalent and the group-effect were taken into account, where the eye, refraction and the group were treated as fixed factors and the subject as a random factor. The results are presented as mean and standard deviation (± SD) for all examined groups, with their corresponding p-values. P < 0.05 was defined as statistically significant.

## Results

100 eyes from 53 controls (31♀ 22♂; 40.2 ± 13.4 years) were compared with 77 eyes from 39 subjects suffering from RP (15♀ 24♂; 45.6 ± 14.7 years) and 18 eyes from 9 participants suffering from CHM (0 ♀ 9 ♂; 40.2 ± 21.2 years).

The molecular characteristics of CHM and RP patients are shown in Table 1 and Supplementary Table 1, respectively.

**Table 1:** Molecular characteristics of included patients with CHM

ID	Gene	Genomic position (hg19)	Nucleotide position	Protein position
CHM_001	*CHM*	NC_000023.10:g.85212858A > T	NM_000390.4:c.940 + 2 T > A	NP_000381.1:p.?
CHM_002	*CHM*	NC_000023.10:g.85212923G > A	NM_000390.4:c.877C > T	NP_000381.1:p.(Arg293Ter)
CHM_003	*CHM*	NC_000023.10:g.85127537G > C	NM_000390.4:c.1770 + 520C > G	NP_000381.1:p.?
CHM_004	*CHM*	NC_000023.10:g.?	Copy number variation involving exons 3–13	NP_000381.1:p.?
CHM_005	*CHM*	NC_000023.10:g.85213877G > A	NM_000390.4:c.808C > T	NP_000381.1:p.(Arg270Ter)
CHM_006	*CHM*	NC_000023.10:g.85218725_85218728del	NM_000390.4:c.649_652del	NP_000381.1:p.(Tyr217HisfsTer14)
CHM_007	*CHM*	NC_000023.10:g.85213886G > A	NM_000390.4:c.799C > T	NP_000381.1:p.(Arg267Ter)
CHM_008	*CHM*	NC_000023.10:g.?	Deletion of exons 1–15	NP_000381.1:p.?
CHM_009	*CHM*	NC_000023.10:g.85211185_85211186insTTCTCCTTGGCCATATAAAGGCCATATAAAGGCCATATAAA	NM_000390.4:c.1138_1139insTTTATATGGCCTTTATATGGCCTTTATATGGCCAAGGAGAA	NP_000381.1:p.(Gln380LeufsTer43)

### Oximetry results

#### Comparison of oxygen saturation values of diseased eyes to controls

In controls, the mean ± SD for A-SO_2_ was 92.69 ± 5.95%, for V-SO_2_ 53.96 ± 9.25% and for A-V SO_2_ 38.73 ± 7.26%. In RP the respective values were: for A-SO_2_ 98.73 ± 9.94%, for V-SO_2_ 62.06 ± 10.59% and for A-V SO_2_ 36.62 ± 8.85%; in CHM these measures were: for A-SO_2_ 77.02 ± 13.40%, for V-SO_2_ 44.33 ± 12.78% and for A-V SO_2_ 32.68 ± 8.27%.

**Fig. 2:**
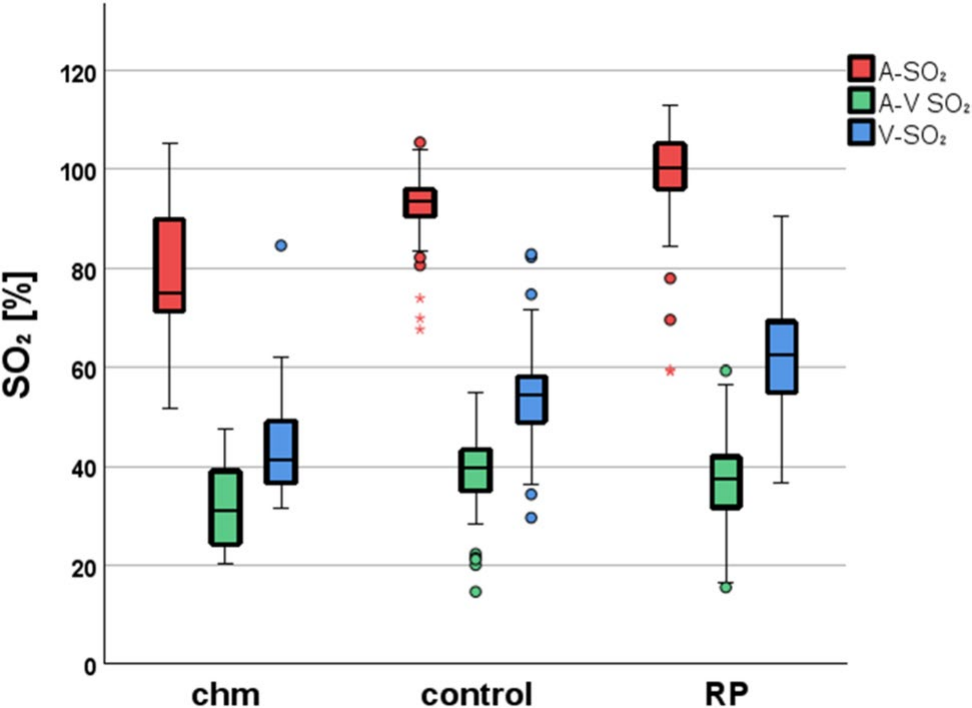
Box Plot analysis illustrates the vessel oxygen saturation of the retinal arterioles (red, A-SO_2_), venules (blue, V-SO_2_), and their difference (green, A-V SO_2_). The ordinate shows the vessel oxygen saturation in percents for all three groups shown on the abscissa (choroideremia = chm, control, retinitis pigmentosa = RP). The upper and lower whiskers of the boxplot present the minimum and maximum value, the upper and lower borders of the box represent the 25th and 75th percentile respectively, and the black horizontal bar within the box represents the median value. Data points classified as outliers are marked as circles

In general, the diseased subgroups differed from controls when all oxygen metabolic parameters (A-SO_2_, A-V SO_2_ and V-SO_2_) were taken into account. All variables showed significant changes (*p* < 0.001), however with opposite findings. In RP, as known before, the A-SO_2_ and V-SO_2_ (*p* < 0.001) presented with a statistically significant increment, while the A-V SO_2_ showed a significant decrement (*p* < 0.001). However, in CHM when compared to controls, the A-SO_2_, V-SO_2_ and A-V SO_2_ presented a significant decrement (*p* < 0.009, Fig. 2).

#### Comparison of oxygen saturation values of RP and CHM

When comparing the diseased subgroups against each other, the CHM subgroup presented with significantly decreased A-SO_2_ (*p* < 0.001) and V-SO_2_ (*p* < 0.001) in comparison to RP, while both diseased subgroups showed no significant differences in A-V SO_2_ (*p* = 0.15, Fig. 2).

## Discussion

So far, RO analyses have not been reported in patients with CHM. CHM presents with a progressive degeneration of the choroid, the RPE, and the photoreceptor layers. Focal peripheral choroidal atrophy can be observed in the early stages of the disease, corresponding to areas of VF loss progressing centripetally. While genetic and biochemical alterations have been well studied in CHM, the pathogenetic sequence and interdependence of structural decay have not been fully elucidated yet [13, 20].

In our study, we found significant changes in the retinal oxygen metabolism of CHM patients when compared to controls by means of significantly lower arterial and venous hemoglobin oxygen saturation and reduced oxygen consumption in the diseased retina. However, when comparing those results to a well-studied IRD (RP), we observed opposing findings with even lower arterial and venous oxygen saturation while both diseases presented with a significantly reduced oxygen consumption.

Local retinal oxidative stress as well as systemic effects seem to play a role in cones’, rods’ and inner retinal cells’ apoptosis in the pathogenic sequence of RP [21, 22]. Numerous studies have successfully demonstrated the fundamental role of oxygen metabolism in the pathogenesis of IRDs with a severely decreased metabolic function in RP: increased arteriolar and venular oxygen saturation levels and reduced arterio-venular differences were measured in RP patients [2–7]. It is hypothesized that the reduced A-V difference is a consequence of photoreceptor cell loss [2–7]. Moreover, as a result of a compromised blood-retina barrier, the influx of proteinaceous and oxygen metabolites from the underlying choriocapillaris aggravates the hyperoxygenated state of the extracellular space, thus amplifying the deleterious effects of oxidative metabolites [23, 24]. The higher extracellular oxygen levels are believed to induce a vasoconstrictive stimulus, leading to reduced flow in retinal arteries and imbalanced retinal hemodynamics [25]. In this study, we were able to confirm established vascular retinal oxygen changes in RP patients compared to controls.

Opposing to this, CHM is associated with more profound perturbation of the choroid, which may result in an insufficient availability of oxygen in retinal tissues compared to controls or RP patients. Our study seems to support this hypothesis: low arterial and venous saturations appear to represent the low oxygen supply to the retinal tissue from an impaired choroidal vasculature. The insufficient supply of oxygen, theoretically needed to maintain adequate retinal function, might contribute to the degeneration of photoreceptors and the RPE. In addition, relative hypoxia could induce activation and hypertrophy of Müller cells [26], which could partially explain the retinal thickening observed during the course of the disease [27]. We hypothesize the low retinal arterial and venous saturations to be the consequence of compensation mechanisms for the lack of choroidal supply and, therefore, leading to lower O_2_ saturation values in the main retinal vessels. To try to explain the decrease of about 15% in parapapillary retinal arterial saturation in CHM we would like to refer to previously hypothesized potential mechanisms, including oxygen diffusion through the vessel walls of the retinal arterioles and the close proximity of the central retinal artery and the central retinal vein within the optic nerve head [28]. This intimate vicinity may lead to oxygen countercurrent exchange between these vessels. Moreover, if at some point the retinal venous saturation decreases further in CHM, this would accentuate the gradient within the optic nerve head and further enhance this mechanism.

Of note, the possibility of confounding of results due to differences in fundus pigmentation between subjects must be acknowledged. Although it was found that a darker fundus pigmentation can lead to lower oxygen saturation values [29], we found higher oxygen saturation values in healthy and RP fundi. In contrast, subjects with depigmented fundi like in CHM presented with lower saturation values. We therefore believe that our results are a reflection of the underlying pathophysiological changes while acknowledging that a potential bias is still possible.

It is believed that choroidal thinning in CHM has its origin in a dual mechanism, similar to the alterations observed in dry age-related macular degeneration [30, 31]. First, there seems to be a thinned choroid at baseline in CHM, followed by a continuous and progressive choroidal thinning as the overlying RPE loses its function [28]. As controversies persist with regard to the exact interdependence of pathophysiological mechanisms in CHM [32], RO might gain increasing relevance to help unveil early vascular and metabolic changes in the retina of these patients. While recent data suggest RPE degeneration to be an early pathogenic factor in CHM with the involution of photoreceptors and secondary choroidal atrophy [33], an exact comprehension of the pathogenesis of this incurable IRD is essential as emerging treatments will aim to tackle the correct order of pathological events. Further prospective large-scale retinal oximetry studies in choroideremia patients including measurements of retinal and choroidal blood flow [34, 35] could help to shed more light on the precise sequential anomalies of CHM.

## Supplementary Information

Below is the link to the electronic supplementary material.

Supplementary file1 (DOCX 22 KB)
