# Supplementary material for: Retinal oxygen metabolic function in choroideremia and retinitis pigmentosa
Source: Graefes Arch Clin Exp Ophthalmol. 2024 Oct 12;263(2):379–85. doi: 10.1007/s00417-024-06659-8 (PMC11868133; doi:10.1007/s00417-024-06659-8)
Supplement: Supplementary file 1 — Supplementary file1 (DOCX 22 KB) [file 417_2024_6659_MOESM1_ESM.docx]

| **Supplementary Table 1. Molecular characteristics of included patients with retinitis pigmentosa** | | | | | | | |
| --- | --- | --- | --- | --- | --- | --- | --- |
| **ID** | **Gene** | **Genomic position 1 (hg19)** | **Nucleotide position 1** | **Protein position 1** | **Genomic position 2** | **Nucleotide position 2** | **Protein position 2** |
| RP001 | *IFT140* | NC_000016.9:g.1637962C>T | NM_014714.4:c.874G>A | NP_055529.2:p.(Val292Met) | NC_000016.9:g.1565635_1571303dup | Duplication of exons 27-30 | NP_055529.2:p.Tyr1152_Thr1394dup |
| RP002 | Unknown |  |  |  |  |  |  |
| RP003 | *EYS* | NC_000006.11:g.64472482del | NM_001142800.2:c.7943del | NP_001136272.1:p.(Thr2648LysfsTer34) | NC_000006.11:g.? | Deletion of exon 1 | NP_001136272.1:p.? |
| RP004 | Unknown |  |  |  |  |  |  |
| RP005 | *EYS* | NC_000006.11:g.64431133_64431136del | NM_001142800.2:c.8793_8796del | NP_001136272.1:p.(Gln2931HisfsTer43) | NC_000006.11:g.64431133_64431136del | NM_001142800.2:c.8793_8796del | NP_001136272.1:p.(Gln2931HisfsTer43) |
| RP006 | *RPGR* | NC_000023.10:g.38145417_38145436dup | NM_001034853.2:c.2819_2838dup | NP_001030025.1:p.(Glu947LysfsTer149) |  |  |  |
| RP007 | *PRPF31* | NC_000019.9:g.54627148_54627180dup | NM_015629.4:c.548_580dup | NP_056444.3:p.(Glu183_Met193dup) |  |  |  |
| RP008 | *USH2A* | NC_000001.10:g.215824058G>T | NM_206933.4:c.14219C>A | NP_996816.3:p.(Ala4740Asp) | NC_000001.10:g.216420437del | NM_206933.4:c.2299del | NP_996816.3:p.(Glu767SerfsTer21) |
| RP009 | Unknown |  |  |  |  |  |  |
| RP010 | Unknown |  |  |  |  |  |  |
| RP011 | *CRB1* | NC_000001.10:g.197396856A>T | NM_201253.2:c.2401A>T | NP_957705.1:p.(Lys801Ter) | NC_000001.10:g.197410093C>G | NM_201253.2:c.3879-1203C>G | NP_957705.1:p.? |
| RP012 | Unknown |  |  |  |  |  |  |
| RP013 | Unknown |  |  |  |  |  |  |
| RP014 | *RHO* | NC_000003.11:g.129247725T>G | NM_000539.3:c.149T>G | NP_000530.1:p.(Leu50Arg) |  |  |  |
| RP015 | *RPGR* | NC_000023.10:g.38145751del | NM_001034853.2:c.2501del | NP_001030025.1:p.(Glu834GlyfsTer255) |  |  |  |
| RP016 | *EYS* | NC_000006.11:g.65655779C>T | NM_001142800.2:c.2288G>A | NP_001136272.1:p.(Trp763Ter) | NC_000006.11:g.? | Deletion of exons 34-36 | NP_001136272.1:p.? |
| RP017 | *USH2A* | NC_000001.10:g.215814065G>A | NM_206933.4:c.14803C>T | NP_996816.3:p.(Arg4935Ter) | NC_000001.10:g.216420437del | NM_206933.4:c.2299del | NP_996816.3:p.(Glu767SerfsTer21) |
| RP018 | *POMGNT1* | NC_000001.10:g.46661556G>T | NM_017739.4:c.461C>A | NP_060209.4:p.(Pro154His) | NC_000001.10:g.46660618G>A | NM_017739.4:c.550C>T | NP_060209.4:p.(His184Tyr) |
| RP019 | Unknown |  |  |  |  |  |  |
| RP020 | Unknown |  |  |  |  |  |  |
| RP021 | Unknown |  |  |  |  |  |  |
| RP022 | *PHYH* | NC_000010.10:g.13330508T>C | NM_006214.4:c.530A>G | NP_006205.1:p.(Asp177Gly) | NC_000010.10:g.13330508T>C | NM_006214.4:c.530A>G | NP_006205.1:p.(Asp177Gly) |
| RP023 | *RPGR* | NC_000023.10:g.38146111_38146112dup | NM_001034853.2:c.2143_2144dup | NP_001030025.1:p.(Glu716GlyfsTer100) |  |  |  |
| RP024 | *EYS* | NC_000006.11:g.? | Deletion of exon 1 | NP_001136272.1:p.? | NC_000006.11:g.? | Deletion of exon 1 | NP_001136272.1:p.? |
| RP025 | *RHO* | NC_000003.11:g.129252535G>A | NM_000539.3:c.1021G>A | NP_000530.1:p.(Glu341Lys) |  |  |  |
| RP026 | *EYS* | NC_000006.11:g.65301407_65301413del | NM_001142800.2:c.4350_4356del | NP_001136272.1:p.(Ile1451ProfsTer3) | NC_000006.11:g.65301407_65301413del | NM_001142800.2:c.4350_4356del | NP_001136272.1:p.(Ile1451ProfsTer3) |
| RP027 | *SLC24A1* | NC_000015.9:g.65916899C>T | NM_004727.3:c.481C>T | NP_004718.1:p.(Gln161Ter) | NC_000015.9:g.65916899C>T | NM_004727.3:c.481C>T | NP_004718.1:p.(Gln161Ter) |
| RP028 | Unknown |  |  |  |  |  |  |
| RP029 | *EYS* | NC_000006.11:g.64940493C>T | NM_001142800.2:c.6416G>A | NP_001136272.1:p.(Cys2139Tyr) | NC_000006.11:g.? | Deletion of exon 1 | NP_001136272.1:p.? |
| RP030 | *EYS* | NC_000006.11:g.65622488G>A | NM_001142800.2:c.2530C>T | NP_001136272.1:p.(Gln844Ter) | NC_000006.11:g.64940493C>T | NM_001142800.2:c.6416G>A | NP_001136272.1:p.(Cys2139Tyr) |
| RP031 | *EYS* | NC_000006.11:g.65622488G>A | NM_001142800.2:c.2530C>T | NP_001136272.1:p.(Gln844Ter) | NC_000006.11:g.64940493C>T | NM_001142800.2:c.6416G>A | NP_001136272.1:p.(Cys2139Tyr) |
| RP032 | *USH2A* | NC_000001.10:g.216595626del | NM_206933.4:c.55del | NP_996816.3:p.(Met19CysfsTer2) | NC_000001.10:g.215940063G>T | NM_206933.4:c.11007C>A | NP_996816.3:p.(Ser3669Arg) |
| RP033 | *SLC24A1* | NC_000015.9:g.65916899C>T | NM_004727.3:c.481C>T | NP_004718.1:p.(Gln161Ter) | NC_000015.9:g.65916899C>T | NM_004727.3:c.481C>T | NP_004718.1:p.(Gln161Ter) |
| RP034 | *PRPF31* | NC_000019.9:g.54627148_54627180dup | NM_015629.4:c.548_580dup | NP_056444.3:p.(Glu183_Met193dup) |  |  |  |
| RP035 | *PRPF31* | NC_000019.9:g.54621704_54621705del | NM_015629.4:c.46_47del | NP_056444.3:p.(Glu16ArgfsTer41) |  |  |  |
| RP036 | Unknown |  |  |  |  |  |  |
| RP037 | *CLRN1* | NC_000003.11:g.150645894A>C | NM_052995.2:c.300T>G | NP_443721.1:p.(Tyr100Ter) | NC_000003.11:g.150645894A>C | NM_052995.2:c.300T>G | NP_443721.1:p.(Tyr100Ter) |
| RP038 | *USH2A* | NC_000001.10:g.216538426A>T | NM_206933.4:c.653T>A | NP_996816.3:p.(Val218Glu) | NC_000001.10:g.215802356del | NM_206933.4:c.15322del | NP_996816.3:p.(Arg5108GlyfsTer6) |
| RP039 | Unknown |  |  |  |  |  |  |
| Unknown= not genetically analyzed or genetically analyzed but without molecular diagnosis | | | | | | | |
